# Light Sensitivity in Myasthenia Gravis: Clinical Characteristics and Impact on Quality of Life

**DOI:** 10.1002/mus.28386

**Published:** 2025-03-05

**Authors:** Eleonora Sabatelli, Luca Bonagura, Silvia Falso, Sofia Marini, Claudia Papi, Lucia Campetella, Michela Myriam De Maio, Raffaele Iorio

**Affiliations:** ^1^ Department of Neuroscience Università Cattolica del Sacro Cuore Rome Italy; ^2^ Neurology Unit Fondazione Policlinico Universitario A. Gemelli IRCCS Rome Italy

**Keywords:** disease severity, light sensitivity, myasthenia gravis, ocular symptoms, quality of life

## Abstract

**Introduction/Aims:**

Light sensitivity is occasionally reported in myasthenia gravis patients, yet its prevalence, clinical characteristics, and impact on disease severity and quality of life remain insufficiently explored. This study aimed to evaluate the frequency, clinical characteristics, and the correlation of light sensitivity with myasthenia gravis severity and quality of life in patients.

**Methods:**

Myasthenia gravis patients consecutively admitted to Fondazione Policlinico Universitario A. Gemelli in Rome between March and October 2023 were considered for the study. A demographically matched group of healthy controls was also included. Patients and controls completed the Visual Light Sensitivity Questionnaire‐8 (VLSQ‐8). The patient cohort additionally received the Quantitative Myasthenia Gravis (QMG) score, the Myasthenia Gravis Activities of Daily Living (MG‐ADL) scale, and the Myasthenia Gravis Quality‐of‐Life‐15 (MG‐QoL‐15) questionnaires.

**Results:**

A total of 92 patients and 75 healthy controls participated. Light sensitivity was significantly more frequent among patients (36%, 33/92) than among controls (4%, 3/75). Patients with light sensitivity exhibited higher QMG (*p* < 0.0001), MG‐ADL (*p* < 0.0001), and a decreased quality of life with higher MG‐QoL‐15 (*p* = 0.0003) scores than those without light sensitivity. Notably, light sensitivity severity positively correlated with diminished quality of life.

**Discussion:**

These findings suggest that light sensitivity may be a feature of myasthenia gravis, with clinically relevant implications for patient well‐being. This underscores the value of screening for and managing light sensitivity in routine myasthenia gravis care. Proactive identification and treatment strategies aimed at light sensitivity may help enhance overall quality of life.

**Abbreviations**
ACHEi: acetylcholinesterase inhibitors
AZA: azathioprine
CI: confidence interval
CS: corticosteroids
Disease duration (years): the number of years since MG diagnosis
EOMG: early onset MG
IS: immunosuppressive drugs
LOMG: late onset MG
LS: light sensitivity
MG: myasthenia gravis
MG‐ADL: Myasthenia Gravis Activities of Daily Living
MG‐ADL ITEM 7: double vision item of the MG‐ADL scale
MG‐ADL ITEM 8: ptosis item of the MG‐ADL scale
MGFA: Myasthenia Gravis Foundation of America
MG‐QoL‐15: Myasthenia Gravis Quality of Life‐15
MGQoL15: Myasthenia Gravis Quality of Life scale, 15‐item version
MM: mycophenolate mofetil
NLS: no light sensitivity
Ocular MGQoL15 ITEM 2: visual quality of life item of the MGQoL15 scale
Ocular QMG ITEM 1: eyelid droop item of the QMG scale
Ocular QMG ITEM 2: extraocular muscle weakness item of the QMG scale
QMG: Quantitative Myasthenia Gravis score
SD: standard deviation
SE: standard error
SLR: simple linear regression
TAMG: thymoma‐associated MG
TOTAL ocular MG‐ADL: total score of the ocular section of the Myasthenia Gravis Activities of Daily Living scale
TOTAL ocular QMG: total ocular score from the Quantitative Myasthenia Gravis scale
VLSQ8: Visual Light Sensitivity Questionnaire
Y: years

## 1 Introduction

Ptosis and diplopia are common clinical manifestations of myasthenia gravis (MG) [1]. With the emergence of novel therapies for MG, there is an increasing emphasis on understanding the full spectrum of MG‐related symptoms and optimizing patient‐centered care [2]. Although some patients with MG report experiencing light sensitivity (LS), this symptom remains relatively underinvestigated [3]. The aim of this study was to assess the prevalence, clinical characteristics, and quality‐of‐life impact of LS in MG patients.

## 2 Methods

### 2.1 Ethics and Study Design

The study, approved by the Ethics Committee of “Fondazione Policlinico Universitario A. Gemelli IRCCS” (Prot. No. 0002692/23, 26/02/2023), followed a prospective design.

### 2.2 Study Population

Patients diagnosed with MG who were consecutively seen as outpatients at the Neurology Unit of Fondazione Policlinico Universitario A. Gemelli in Rome from March to October 2023 and provided informed consent to participate in the study were enrolled. Inclusion criteria were age ≥ 18 years, with a confirmed MG diagnosis based on a combination of clinical examination, serum positivity for AChR antibodies, and electrodiagnostic findings consistent with neuromuscular junction dysfunction. All patients were positive for AChR antibodies and demonstrated clinical evidence of involvement of extraocular muscles, defined as the presence of ptosis or diplopia. Additionally, a group of sex‐ and age‐matched healthy controls (HCs), without a diagnosis of MG, was recruited from individuals accompanying patients at the outpatient clinic and from hospital staff. Patients and HCs with a history of migraine, ophthalmological disorders, diabetes mellitus, and other neurological or psychiatric conditions were excluded.

MG patients were assessed for the presence and intensity of LS using the Visual Light Sensitivity Questionnaire‐8 (VLSQ‐8) [4]. A score ≥ 3 on the VLSQ‐8 indicated the presence of LS. Patients also underwent standard clinical assessments with the following scales: Quantitative MG (QMG), MG Activities of Daily Living (MG‐ADL), and MG Quality of Life‐15 (MG‐QoL‐15) [5, 6, 7]. HCs were interviewed on‐site to evaluate LS using theVLSQ‐8.

Correlations between LS severity and clinical MG severity, as well as the impact of LS on quality of life and its association with ocular item scores on various scales, were explored. The study also examined the relationship of LS to anti‐cholinesterase therapy intake, disease duration, and different MG clinical subtypes, such as early‐onset generalized seropositive MG (EOMG), including patients with age at clinical onset less than 50 years old; late‐onset generalized seropositive MG (LOMG), including patients with age at clinical onset greater than or equal to 50 years old [8]; and MG associated with thymoma (TAMG).

A subset of MG patients with LS, selected according to their availability and willingness to participate, was re‐evaluated 3 months after their initial assessment. Any clinical or therapeutic changes between the initial (T0) and follow‐up (T1) visits were documented, and correlations between LS severity and various scale scores at T1 were reanalyzed.

### 2.3 Statistical Analysis

Descriptive statistical techniques were used to summarize all variables. Qualitative data were summarized as absolute counts and relative percentages, while quantitative data were represented using means and standard deviations (SD). The Gaussian distribution of the data was verified using the Shapiro–Wilk test, specifically applied to quantitative variables. For between‐group comparisons of qualitative variables at baseline (T0), Fisher's exact test was employed. Owing to the non‐normal distribution of quantitative data at T0, the Mann–Whitney test was used for analysis.

Simple linear regression (SLR) models were employed to assess the relationship between LS severity and scores on various scales at both T0 and T1. The following covariates were included in the linear regression model based on their relevance to the outcome variable (VLSQ8): total MG‐ADL score, ocular items of MG‐ADL (items 7 and 8), total QMG score, ocular QMG scores (items 1 and 2), MGQoL15 score, item 2 of MGQoL15, and disease duration.

Pearson's correlation coefficient was calculated to assess the association between the scores on the VLSQ8 and other clinical scales. Correlations were reported by their strength [9]. Differences between T0 and T1 datasets were analyzed using the Wilcoxon Signed‐Rank Test. All analyses were conducted with a 95% confidence interval (CI) using GraphPad Prism version 10.0 (GraphPad Software Inc., La Jolla, CA) [10].

## 3 Results

### 3.1 Characteristics of the Study Population

A total of 106 patients were enrolled. Fourteen patients were subsequently excluded due to congenital mydriasis [1], cataract with surgical indication [5], maculopathy [1], diabetes mellitus [5], migraine [1], and Sjögren's syndrome [1], leaving a final cohort of 92 patients. The demographics and clinical characteristics are summarized in Table 1. For comparison, 75 demographically matched HCs were included.

**TABLE 1:** Comparative analyses between patients and healthy controls.

Characteristic	MG patients (*n* = 92)	Healthy controls (*n* = 75)	*p*	Difference between medians
Age (y), mean ± SD	57.9 ± 16.99	53.26 ± 17.26	0.084	—
Sex, No. (%)—females	37/92 (42%)	39/75 (52%)	0.126	—
Sex, No. (%)—males	55/92 (58%)	36/75 (48%)	0.126	—
VLSQ8 score, mean ± SD^a^	12.85 ± 6.89	5.0 ± 2	< 0.0001	−6.000

*Note*: Data are presented as number of patients (No) and percentage (%) for categorical variables and as mean ± standard deviation (SD) for continuous variables. Negative values in the median difference indicate higher scores in MG patients group.

Abbreviations: MG: myasthenia gravis, Y: years.

^a^ VLSQ8 score refers to patients and healthy controls with light sensitivity.

### 3.2 LS Frequency and Severity

The prevalence of LS was significantly higher in the patient group at 36% (33/92) compared to 4% (3/75) in HCs (*p* < 0.0001). MG patients with LS had significantly higher scores on the QMG, MG‐ADL, and MG‐QoL‐15 compared to MG patients without LS (Table 2).

**TABLE 2:** Comparative analyses between myasthenia gravis patients with and without light sensitivity.

Characteristic	LS MG patients (*n* = 33)	NLS MG patients (*n* = 59)	*p*	Difference between medians
Age (y), mean ± SD	56.5 ± 19.12	54.93 ± 16.96	0.696	—
Sex, No. (%)—females	12/33 (36%)	27/59 (44%)	0.383	—
Sex, No. (%)—males	21/33 (64%)	33/59 (56%)	0.530	—
EOMG	12/33 (36%)	12/59 (20%)	0.096	—
TAMG	2/33 (6%)	7/59 (12%)	0.367	—
LOMG	19/33 (58%)	40/59 (68%)	0.3204	—
MG‐ADL score, mean ± SD	5.33 ± 3.62	1.4 ± 2.24	< 0.0001	−3.000
QMG score, mean ± SD	6.72 ± 5.39	3.3 ± 2–1	0.0003	−3.000
MG‐QoL15 score, mean ± SD	19.64 ± 16.85	5.81 ± 9.95	< 0.0001	−9.000
AChEi therapy, No. (%)	19/33 (57%)	25/59 (42%)	0.1602	—
MGFA Max^a^‐I	11/33 (33%)	15/59 (25%)	0.4122	—
MGFA Max^a^‐IIa	7/33 (21%)	5/59 (8%)	0.637	—
MGFA Max^a^‐IIb	4/33 (12.12)	17/59 (29%)	0.0662	—
MGFA Max^a^‐IIIa	7/33 (21.21)	8/59 (13%)	0.788	—
MGFA Max^a^‐IIIb	3/33 (9.1)	8/59 (13%)	0.368	—
MGFA Max^a^‐IVa	1/33 (3%)	1/59 (2%)	0.952	—
MGFA Max^a^‐IVb	0/33 (0)	4/59 (7%)	0.139	—
MGFA Max^a^‐V	0/33 (0)	1/59 (2%)	0.492	—
Immunosuppressive therapy—CS	16/33 (49%)	26/59 (4%)	0.206	—
Immunosuppressive therapy—AZA	1/33 (3%)	1/59 (2%)	0.952	—
Immunosuppressive therapy—CS + AZA	1/33 (3%)	5/59 (8%)	0.351	—
Immunosuppressive therapy—MM	1/33 (3%)	2/59 (3%)	0.931	—
Immunosuppressive therapy—CS + MM	1/33 (3%)	2/59 (3%)	0.931	—
None	13/33 (39%)	23/59 (39%)	0.468	—

*Note*: Data are presented as number of patients (No) and percentage (%) for categorical variables and as mean ± standard deviation (SD) for continuous variables. Negative values in the “Difference between medians” column indicate that the median score was higher in the LS (light sensitivity) group compared to the NLS (no light sensitivity) group, reflecting greater disease burden in LS patients.

Abbreviations: ACHEi: acetylcholinesterase inhibitors, AZA: azathioprine, CS: corticosteroids, EOMG: early onset MG, IS: immunosuppressive drugs, LOMG: late onset MG, LS: light sensitivity, MG: myasthenia gravis, MGFA: Myasthenia Gravis Foundation of America, MM: mycophenolate mofetil, NLS: no light sensitivity, TAMG: thymoma associated MG, Y: years.

^a^ MGFA max refers to the highest score achieved in the clinical history of the patient.

SLR indicated a positive, moderate correlation between increased LS severity assessed by the VSLQ8 scale and higher scores on the MG‐QoL‐15 scale (Figure S1), and a weak positive association with the ocular clinical item of the MG‐QoL‐15 scale (*p* = 0.0111). Pearson's correlation revealed a moderate positive correlation between LS severity and total MG‐QoL‐15, ocular MG‐QoL‐15, and item 7 of the MG‐ADL score (Figure S1A and Table 3).

**TABLE 3:** Statistical results of the significant correlations between VLSQ8 and other variables.

	Regression coefficient	SE	Pearson's *r*	*p*	95% confidence interval
MGADL item 7	2.695	1.064	0.41	0.0166	[0.525, 4.866]
MGQoL15	0.283	0.053	0.69	< 0.0001	[0.175, 0.391]
Ocular MGQoL15 item 2	2.074	0.768	0.44	0.0171	[0.508, 3.640]
Disease duration (years)	0.299	0.143	0.35	0.0442	[0.008, 0.591]

We observed no significant correlation between LS prevalence and acetylcholinesterase inhibitor therapy or between LS and the MG subtype, and no significant difference between ocular scores on MG‐ADL and QMG for patients with and without LS. Pearson's correlation revealed a weak positive correlation between LS severity and MG disease duration (Table 3).

In the longitudinal assessment of 10 MG patients with LS, no statistically significant changes were observed between T0 and T1 for VLSQ8, MG‐ADL, MG‐QoL‐15, or QMG scale scores.

## 4 Discussion

Our findings demonstrate that LS is a reported symptom in MG patients and significantly impacts their quality of life, as evidenced by the correlation between LS severity and MG‐QoL15 scores. However, no significant correlation was observed between LS and overall disease severity as measured by MG‐ADL and QMG scores. This highlights that while LS may contribute to the disease burden of MG patients, it does not necessarily align with broader clinical measures of MG severity. The observed correlation between LS severity and disease duration warrants further exploration. While this association suggests LS may persist or even worsen over time, the cross‐sectional nature of our study prevents conclusions about causality. Longitudinal studies with extended follow‐up are necessary to determine whether LS evolves with disease progression.

Interestingly, a modest correlation was found between LS severity and ocular muscle weakness, as reflected in the ocular MG‐QoL15 and MG‐ADL item 7 scores. This finding aligns with previous hypotheses that LS may arise as a compensatory mechanism for ocular symptoms such as ptosis [3]. However, our data did not show significant differences in ocular scores between LS‐positive and LS‐negative patients, highlighting the need for further research on this question.

The pathophysiological mechanisms underlying LS in MG remain poorly understood. While LS has been hypothesized to stem from neuromuscular dysfunction involving the intrinsic ocular musculature, our study did not directly investigate these mechanisms. Further studies integrating pupillometry, imaging, and functional assessments of ocular muscle activity are required to elucidate the origins of LS in MG.

## 5 Limitations of the Study

LS is a symptom difficult to objectify in a standardized method despite the exclusion of influencing factors. The development of new standardized methods for its assessment should improve its characterization.

The study involved a limited number of patients, in particular those included in the follow‐up period. Future research with larger, more diverse cohorts and longitudinal designs will be critical for validating these findings.

## 6 Conclusions

LS emerges as a possible component of MG clinical manifestations. However, to fully comprehend the precise pathophysiological mechanisms underlying LS in MG, more extensive investigations are needed.

## Author Contributions

**Eleonora Sabatelli:** investigation, writing – original draft, formal analysis, data curation, methodology, validation, project administration, writing – review and editing. **Luca Bonagura:** methodology, data curation, investigation. **Silvia Falso:** data curation, supervision. **Sofia Marini:** data curation, supervision. **Claudia Papi:** supervision, data curation. **Lucia Campetella:** supervision. **Michela Myriam De Maio:** data curation. **Raffaele Iorio:** conceptualization, investigation, writing – review and editing, methodology, supervision, formal analysis, resources, project administration, visualization, funding acquisition, software, validation.

## Disclosure

The work was presented as an abstract at a previous conference (XXIII National Congress of Italian Association of Miology, 8–10 June 2023, Padua, Italy).

## Ethics Statement

All authors confirm to have read the Journal's position on issues involved in ethical publication and affirm that this report is consistent with those guidelines.

## Conflicts of Interest

The authors declare no conflicts of interest.

## Supporting information


Figure S1.

## Data Availability

The data that support the findings of this study are available on request from the corresponding author. The data are not publicly available due to privacy or ethical restrictions.
