# Supplementary figures and images for: Light Sensitivity in Myasthenia Gravis: Clinical Characteristics and Impact on Quality of Life
Source: Muscle Nerve. 2025 Mar 5;71(6):1081–5. doi: 10.1002/mus.28386 (PMC12060601; doi:10.1002/mus.28386)

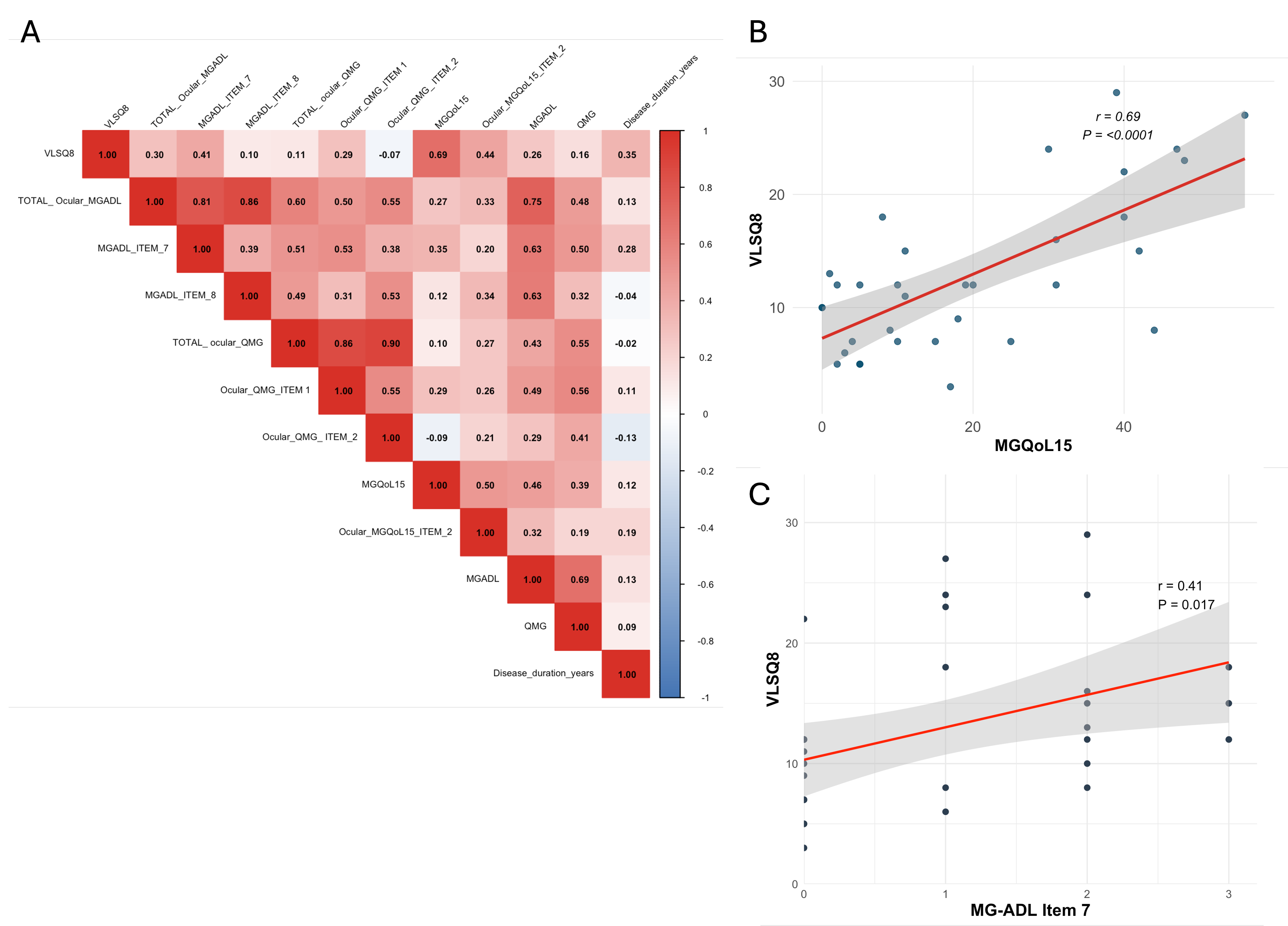

Supplement: Supplementary file 1 — Figure S1. [file MUS-71-1081-s001.tiff]
